# Free-Hand MIS TLIF without 3D Navigation—How to Achieve Low Radiation Exposure for Both Surgeon and Patient

**DOI:** 10.3390/jcm12155125

**Published:** 2023-08-04

**Authors:** Roberto Doria-Medina, Ulrich Hubbe, Christoph Scholz, Ronen Sircar, Johannes Brönner, Herbert Hoedlmoser, Jan-Helge Klingler

**Affiliations:** 1 Department of Neurosurgery, Medical Center, University of Freiburg, 79106 Freiburg, Germany; 2 Mirion Technologies (AWST) GmbH, 81739 Munich, Germany

**Keywords:** spine surgery, minimally invasive surgery, transforaminal interbody fusion, conventional fluoroscopy, computer assisted navigation, radiation exposure

## Abstract

Background: Transforaminal lumbar interbody fusion (TLIF) is one of the most frequently performed spinal fusion techniques, and this minimally invasive (MIS) approach has advantages over the traditional open approach. A drawback is the higher radiation exposure for the surgeon when conventional fluoroscopy (2D-fluoroscopy) is used. While computer-assisted navigation (CAN) reduce the surgeon’s radiation exposure, the patient’s exposure is higher. When we investigated 2D-fluoroscopically guided and 3D-navigated MIS TLIF in a randomized controlled trial, we detected low radiation doses for both the surgeon and the patient in the 2D-fluoroscopy group. Therefore, we extended the dataset, and herein, we report the radiation-sparing surgical technique of 2D-fluoroscopy-guided MIS TLIF. Methods: Monosegmental and bisegmental MIS TLIF was performed on 24 patients in adherence to advanced radiation protection principles and a radiation-sparing surgical protocol. Dedicated dosemeters recorded patient and surgeon radiation exposure. For safety assessment, pedicle screw accuracy was graded according to the Gertzbein–Robbins classification. Results: In total, 99 of 102 (97.1%) pedicle screws were correctly positioned (Gertzbein grade A/B). No breach caused neurological symptoms or necessitated revision surgery. The effective radiation dose to the surgeon was 41 ± 12 µSv per segment. Fluoroscopy time was 64 ± 34 s and 75 ± 43 radiographic images per segment were performed. Patient radiation doses at the neck, chest, and umbilical area were 65 ± 40, 123 ± 116, and 823 ± 862 µSv per segment, respectively. Conclusions: Using a dedicated radiation-sparing free-hand technique, 2D-fluoroscopy-guided MIS TLIF is successfully achievable with low radiation exposure to both the surgeon and the patient. With this technique, the maximum annual radiation exposure to the surgeon will not be exceeded, even with workday use.

## 1. Introduction

Transforaminal lumbar interbody fusion (TLIF) is one of the most frequently performed stabilization procedures for the treatment of degenerative lumbar disease [1]. The minimally invasive TLIF (MIS TLIF) technique was first described by Foley and associates in 2001 and has become an established mainstay procedure in spine surgery due to the lower tissue trauma, lower blood loss, shorter operation times, decreased postoperative pain, lower required narcotic use, faster recovery, shorter hospital stay, and good outcomes in patients with obesity [2,3,4,5,6,7,8,9]. However, since anatomical structures cannot be directly visualized, MIS TLIF necessitates intraoperative radiographs based on ionizing radiation, which poses a potential health risk for both the surgical team and the patient which has been reported to exceed that of open TLIF procedures [3,5,10,11,12,13,14,15,16,17].

It has been widely recognized that low radiation exposure to the surgeon can only be ensured by using computer-assisted navigation (CAN), as the medical staff can leave the operating room during intraoperative image acquisition. However, the acquisition of an intraoperative 3D dataset of multiple X-rays from a 3D C-arm or from intraoperative computed tomography (iCT) causes considerably higher radiation exposure to the patient [18,19]. Moreover, most studies on free-hand MIS TLIF with conventional (2D) fluoroscopy report comparatively high radiation exposure to the surgeon [20].

In a randomized controlled trial, we previously reported low radiation exposures to the surgeon during monosegmental MIS TLIF with both 3D-fluoroscopy-based CAN and 2D-fluoroscopy, but with significant higher radiation exposure to the patient using 3D-fluoroscopy-based CAN [18]. Based on these results, the aim of this work is to present a radiation-sparing free-hand technique for MIS TLIF using 2D-fluoroscopy and to report on surgical pearls and pitfalls.

## 2. Materials and Methods

This prospective study includes 24 patients who underwent monosegmental or bisegmental MIS TLIF procedures with 2D-fluoroscopy. They are a subset of patients from a primary study whose protocol, along with inclusion and exclusion criteria, has been published elsewhere [21].

Unused dosemeters for every operation were placed at various points on the patient and the surgeon [18,21]. All dosemeters were read at the individual monitoring service of the Helmholtz Zentrum München (German Research Center for Environmental Health, Munich, Germany), now Mirion Technologies (AWST) GmbH, with the assumption of a lower detection limit of 44 µSv for film dosemeters according to GUM (Guide to the Expression of Uncertainty in Measurement)-based uncertainty models [22]. A mobile 3D C-arm device with a flat panel detector (Ziehm Vision FD Vario 3D, Ziehm Imaging, Nuremberg, Germany) was used for 2D-fluoroscopy; it consisted of single fluoroscopic images only. The mobile C-arm device used for all procedures was set on automatic exposure control with corresponding kilovolt and milliampere values of varying magnitudes. Radiation exposure in time (seconds), number of images, total radiation dose (µSv), and dose–area product (cGycm^2^) were collected. The radiation exposure was collected from the patient’s neck, chest, and umbilical area. The effective radiation dose experienced by the surgeon was calculated using the following established algorithm from the 2007 Recommendations of the International Commission on Radiological Protection (ICRP):

E = 0.11 × *Ha* + 0.89 × *Hb*,

(*Ha* = measured unprotected at the thyroid at 1-cm of depth, *Hb* = measured protected on the chest at 1-cm of depth) [23]. The surgeon wore protective gear (two-piece lead apron protecting the front and back, reaching from the torso to the knee; a thyroid lead collar; and protective goggles).

To evaluate the satisfactory pedicle screw placement of our free-hand radiation sparing technique, the Gertzbein–Robbins classification was used to assess pedicle screw accuracy on a postoperative CT scan by measuring the pedicle breach on a 5-graded scale with 2 mm increments [24]. All breaches were documented and classified, with breaches of more than 2 mm considered relevant, as is common practice [25].

The local ethics committee approved the study (number 431/12). The study was registered with the German Clinical Trials Register (www.drks.de; DRKS00004514, registered on 8 November 2012, accessed on 1 May 2023). Written informed consent was obtained from each participant before inclusion in the primary study and treatment.

During the MIS TLIF procedures, the surgical team strictly adhered to the following advanced radiation protection principles and radiation-sparing surgical protocol.

### 2.1. Advanced Radiation Protection Principles

1. Wearing protective gear (two-piece lead apron, thyroid lead collar, and protective goggles);
2. Optimal use of a beam collimator whenever possible;
3. Minimized distance from the patient to the flat panel detector;
4. Maximized staff distance from the patient as source of Compton scattering;
5. Removal of the hand from the X-ray beam when holding an instrument is necessary (use of surgical clamp to increase distance);
6. No continuous fluoroscopy.

### 2.2. Radiation-Sparing Surgical Protocol

1. Precise preoperative planning of screw trajectories and screw dimensions

**Figure 1:**
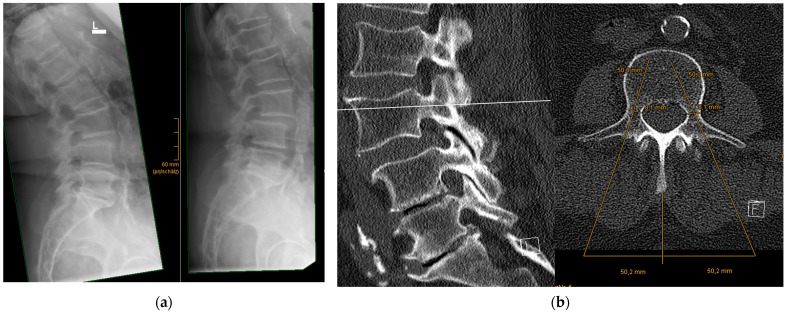
(**a**) Exemplary case of a 79-year-old woman with unstable spondylolisthesis at L3–L4 causing intractable pain unresponsive to conservative treatment. Consequently, MIS TLIF of L3–L4 was indicated. (**b**) The preoperative CT-scan in sagittal (left) and axial (right) cross sections, displaying the measured trajectory, convergence angles, and screw dimensions (here, 6.5 mm × 50 mm), as well as the lateral incision point of 5 cm.

Screw trajectories and their convergence angles and screw dimensions should be anticipated in preoperative CT/MRI scans before surgery. To determine the position of a skin incision, the lateral incision point should be measured (Figure 1).

2. Using a metal template for entry point identification

**Figure 2:**
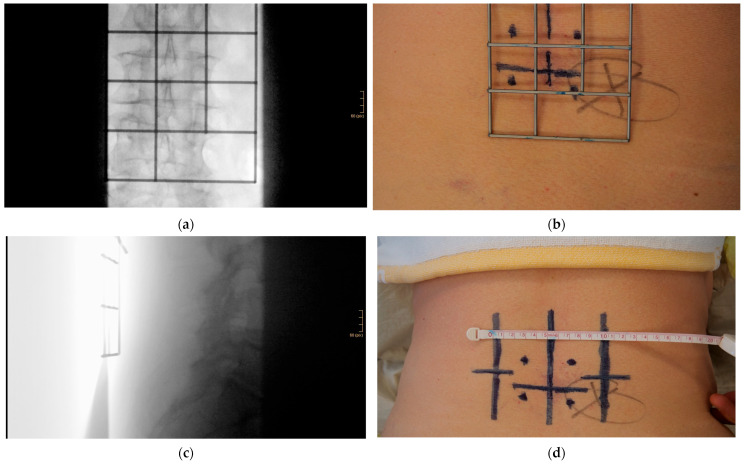
(**a**) A metal template is used as a localization tool to identify the correct segment as well as the involved pedicles and intervertebral discs with one single ap radiographic image. (**b**) The midline, pedicles, and intervertebral disc are marked. (**c**) One additional lateral fluoroscopic image depicts the plane of the paramedian transverse skin incisions, usually at about the level of the upper pedicle. (**d**) The laterality of the skin incision is marked by two longitudinal lines (here, 5 cm paramedian). The required metal grid had already been removed in the photo. In this study, the Sextant II system (Medtronic) was primarily used. For other minimally invasive screw–rod systems with rod insertion through the same skin incision as the pedicle screws, a longitudinal skin incision may be considered meaningful. Note the constant application of beam collimation in each fluoroscopic image.

The patient is placed in prone position on a radiolucent table, allowing for fluoroscopic images to be captured in anterior–posterior (ap) and lateral projection. By using a metal marking template, one single-shot ap fluoroscopic image is usually sufficient to mark the involved pedicles, the intervertebral disc, the midline, and the paramedian screw insertion points on the skin. One lateral single-shot fluoroscopic image is used to mark the plane of the paramedian skin incisions, usually 3 to 4 cm in length. Beam collimation should be applied whenever possible (Figure 2).

3. Jamshidi needle positioning and K-wire placement

**Figure 3:**
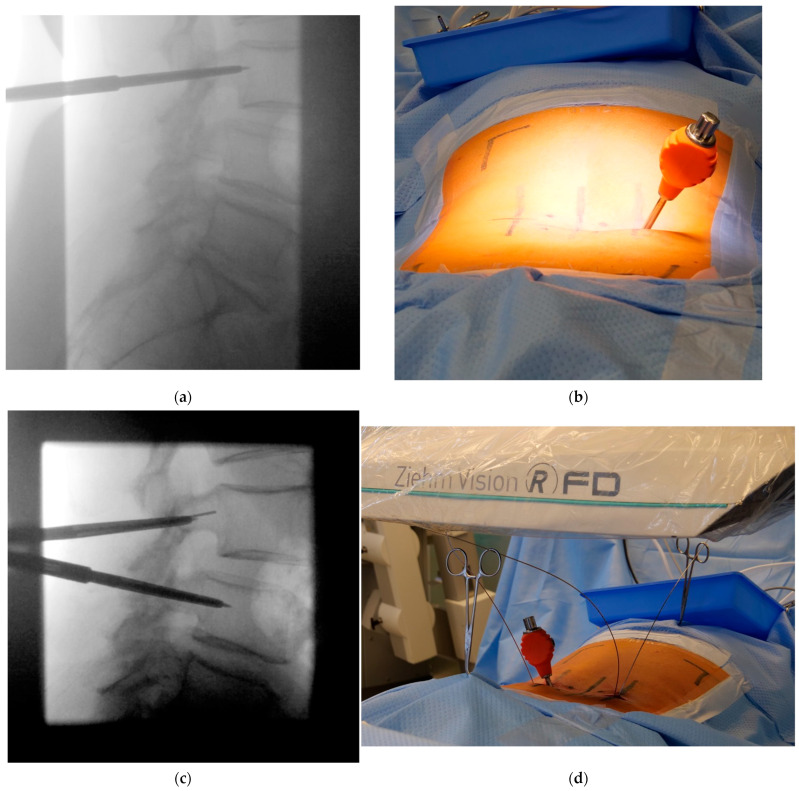
(**a**) Pediculation with the Jamshidi using tactile feedback under sparse lateral radiographic control followed by K-wire insertion. (**b**) The corresponding convergence angle must be followed. It is not necessary to hold the instruments while performing the fluoroscopic imaging. (**c**) To save on fluoroscopic images, Jamshidi needles can be inserted on both sides simultaneously—preferably into different vertebral bodies to allow for radiographic differentiation. (**d**) The Jamshidi needle does not have to be held during fluoroscopic imaging; thus, standing behind a mobile radiation protection wall is possible.

With tactile feedback and sparse lateral fluoroscopic imaging, a Jamshidi needle enters via the paramedian skin incision at the ideal entry point between the transverse process and the lateral facet joint. The corresponding convergence angle, which has already been measured, must be followed if necessary by verification with a view from the patient’s foot. Collimated single lateral fluoroscopic images confirm correct Jamshidi needle advancement into the posterior third of the corresponding vertebral body, which is followed by the insertion of a Kirschner (K) wire. To save on fluoroscopic images, Jamshidi needles can be inserted on both sides simultaneously, preferably into different vertebral bodies, to allow for radiographic differentiation. The Jamshidi needle does not have to be held during fluoroscopic imaging; thus, all staff, including the surgeon, may increase their distance from the radiation source and step behind a mobile radiation protection wall if available (Figure 3).

4. Confirmation of strictly transpedicular K-wire positioning

**Figure 4:**
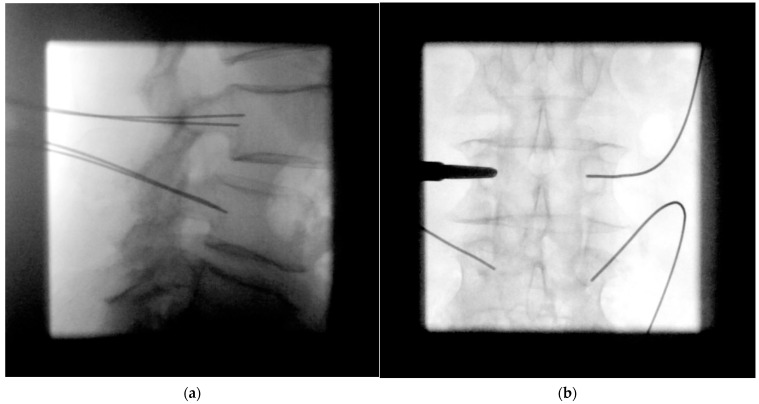
(**a**) In the lateral fluoroscopic image, the K-wires are now retracted to the level of the posterior wall of the vertebral body. (**b**) In the subsequent ap fluoroscopic image, a strictly intrapedicular K-wire placement should be confirmed by not exceeding the medial edge of the pedicle.

After placement of all K-wires, they are withdrawn to the posterior wall of the vertebral body under further lateral fluoroscopic control, followed by an ap fluoroscopic image to verify their correct transpedicular trajectories and to avoid medial pedicle perforation by the subsequently inserted screw (Figure 4). The K-wires are then advanced to secure the trajectories.

5. Ipsilateral placement of a tubular non-expandable retractor

**Figure 5:**
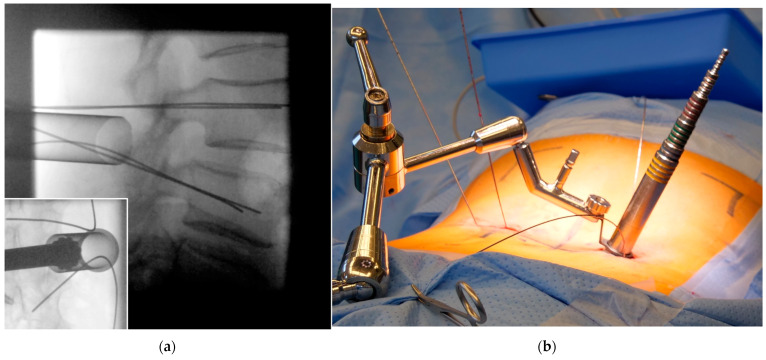
(**a**) The TLIF approach is obtained via dilation and subsequent insertion of a non-expandable tubular retractor 20 mm in diameter onto the ipsilateral L3–L4 facet joint under lateral fluoroscopic guidance. This is followed by ap fluoroscopic control to confirm adequate convergence (insertion). (**b**) The tubular retractor is positioned between the L3 and L4 pedicles to prevent injury to the pedicles during access preparation. The K-wires are advanced further into the vertebral bodies. Note that the K-wires are held away with clamps.

The ipsilateral facet joint of the TLIF approach is identified through the initial skin incision with the smallest tubular dilator under lateral fluoroscopic imaging (the ipsilateral K-wires are held away with clamps cranially and caudally). The access is widened with progressively larger dilators before the definitive 20 mm diameter non-expandable tubular retractor (METRx^®^, Medtronic, Minneapolis, MN, USA) is installed and controlled by one ap fluoroscopic image if necessary (Figure 5).

6. Pedicle screw insertion

**Figure 6:**
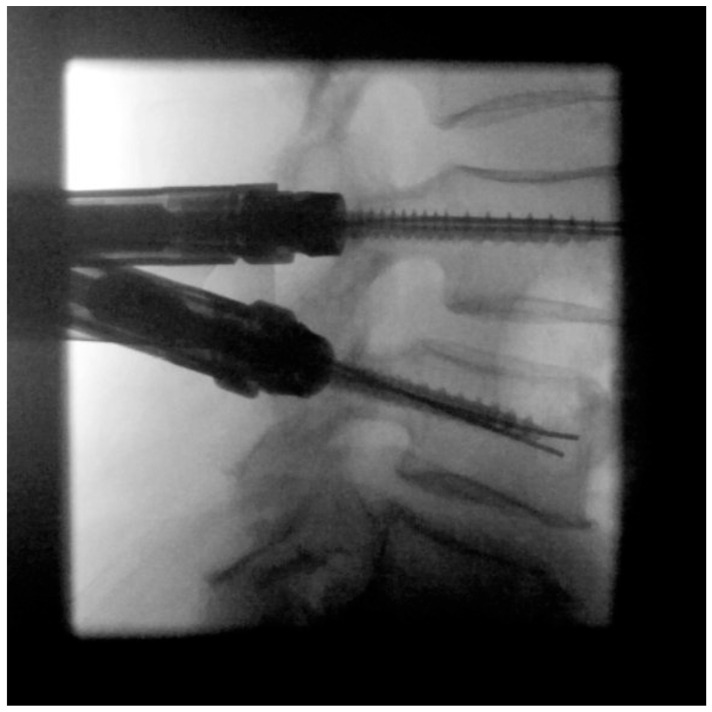
The contralateral pedicle screws are inserted to later maintain the distraction of a potentially reduced intervertebral disc space.

The pedicle screws contralateral to the TLIF approach are now ready for insertion to later maintain distraction of the disc space if necessary (Figure 6).

The correct pedicle screw depth can be noted by an increase in resistance, indicating that the head of the pedicle screw has reached the posterior bone structures. Imaging is rarely necessary at this point.

7. Facetectomy, decompression, discectomy, and distraction

**Figure 7:**
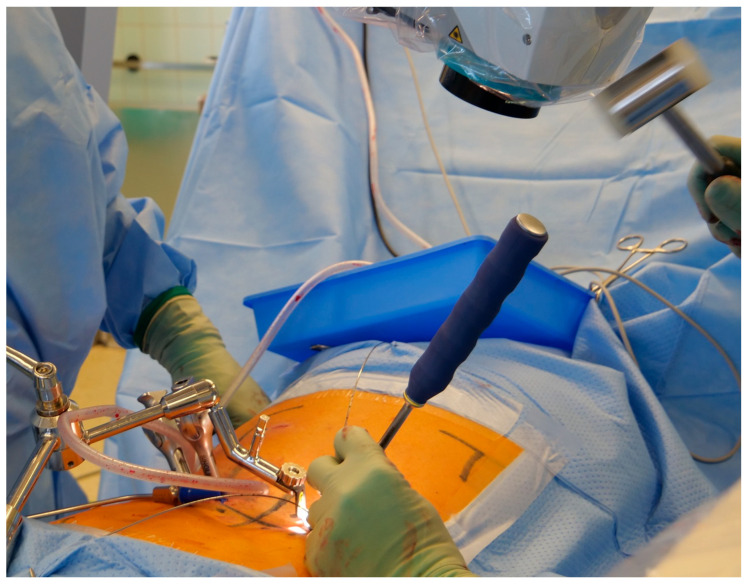
Under microscopic view, ipsilateral facetectomy of L3–L4 is performed (here with a chisel) and the autologous bone is harvested. The nerve structures can be decompressed up to the contralateral recess (under the increasing convergent position of the tubular retractor). Fluoroscopic imaging is usually not required for these steps. Distraction can be maintained via the contralateral screw–rod system.

Under microscopic magnification, ipsilateral facetectomy; ipsilateral or, if necessary, bilateral osteoligamentous decompression of the spinal canal; and discectomy follow without any further need for fluoroscopic images through the tube (Figure 7).

After discectomy and shaving of the vertebral body endplates, progressive distraction of the motion segment is achieved. Distraction, especially of an originally collapsed disc space, which allows for both lordosis and enlargement and, thus, indirect decompression of both neuroforamina, are of great importance. If the intervertebral space is consumed and highly compromised, it may be necessary to initially insert a chisel into the intervertebral disc space under lateral fluoroscopic control. The re-collapse of the disc space before insertion of the definitive intervertebral cage can be avoided by distraction via the already-placed contralateral pedicle screws.

8. Cage placement under lateral radiographic control

**Figure 8:**
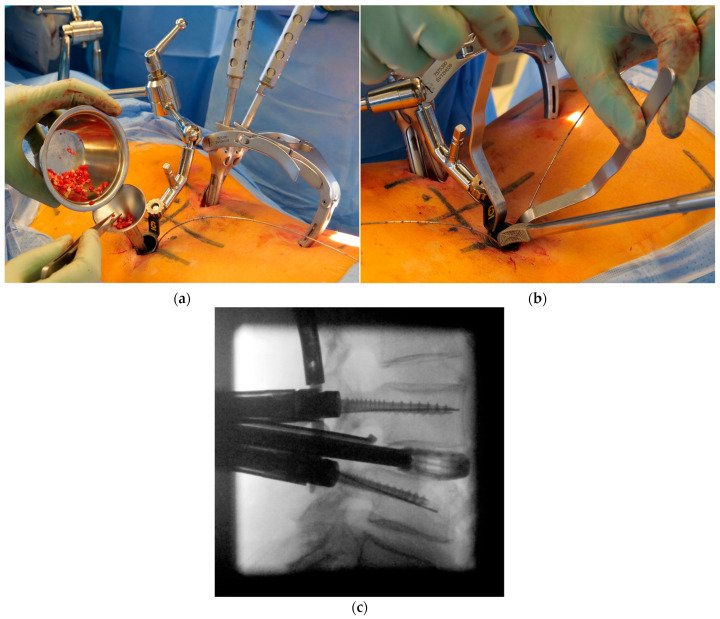
(**a**) The minced autologous bone is inserted and compacted in the intervertebral space. Fluoroscopic imaging is usually not required for these steps. (**b**) Under protection of the nerve structures, the cage is inserted into the anterior intervertebral space through the tubular device. (**c**) A lateral single-shot fluoroscopic image is taken to confirm correct cage insertion.

Minced autologous bone collected from the facetectomy is deposited and compacted in the intervertebral disc space immediately prior to insertion of the cage. Under protection of the nerve structures, the cage is inserted into the anterior intervertebral space under a lateral single-shot fluoroscopic image (Figure 8).

In case of accidental durotomy, transtubular closure can be accomplished by using special bayonet instruments [26]. If cement augmentation is considered to be necessary, application via perforated screws is performed under successive single lateral fluoroscopic control [27].

9. Ipsilateral screw placement usually without imaging

A similar technique for screw insertion via the inserted K-wires is used as for the contralateral side. Fluoroscopic imaging is usually not required for correct pedicle screw insertion.

10. MIS rod insertion using haptic feedback and no or very sparse lateral radiographic control

**Figure 9:**
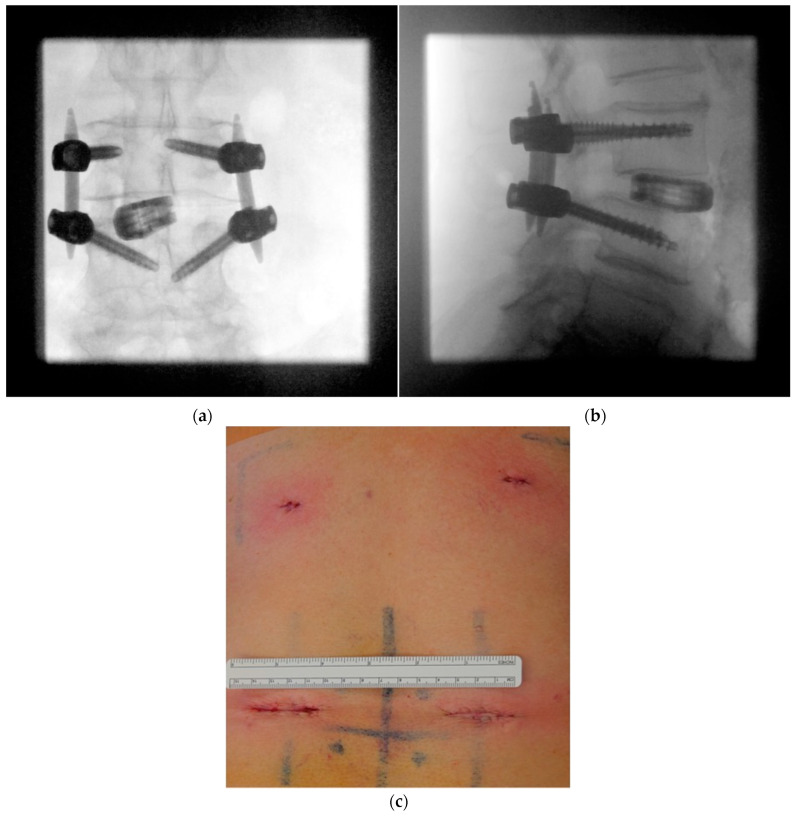
(**a**) The final ap and (**b**) lateral fluoroscopic images confirm correct implant position. (**c**) Demonstration of the minimally invasive wound incisions necessary for the procedure.

Minimally invasive placement of the rod is made with a rod inserter through small skin incisions. Correct placement may be confirmed with a lateral fluoroscopic image, but is usually not necessary. Nuts are tightened and secured with a torque wrench. A final ap and lateral fluoroscopic image confirm the correct placement of the implants before closing the wounds (Figure 9a,b). Figure 9c demonstrates the minimally invasive wound incisions necessary for the procedure.

## 3. Results

### 3.1. Clinical Characteristics

Detailed characteristics of the patients, diagnosis, and surgery are presented in Table 1. Twenty-four patients were included. Three patients received bisegmental stabilization. The female-to-male ratio was equally distributed, and the mean age was 62 years. Pedicle screws were correctly positioned in 99 of 102 (97.1%) cases according to Gertzbein–Robbins grade A or B. No pedicle breach >2 mm (Gertzbein-Robbins grade C: *n* = 1, Gertzbein-Robbins grade D: *n* = 2) caused symptoms, and no revision surgery was necessary during the one-year follow-up period.

### 3.2. Radiation Exposure

Per segment, an average effective dose of 41.4 (±11.7) µSv for the surgeon and average total radiation doses of 65 (±40), 123 (±116), and 823 (±862) µSv for the neck, chest, and umbilical area, respectively, for the patient were measured. The fluoroscopy time was 64 ± 34 s and 75 ± 43 radiographic images per segment were taken. The dose–area product was 526 cGycm^2^. The results are displayed in Table 2.

## 4. Discussion

CAN is still not used by the majority of spine surgeons [28,29,30]. Consequently, MIS TLIF is still performed with 2D-fluoroscopy by several spine surgeons. This paper illustrates the surgical technique, intraoperative handling of ionizing radiation, and use of different radiation protection measures in MIS TLIF on the basis of dosemetric data from a prospective study [18]. This illustration not only serves as a guideline for a free-hand radiation-sparing surgical technique for MIS TLIF, but is also intended to raise awareness of intraoperatively used ionizing radiation, and thus also to promote surgical techniques of other procedures and the use of all available radiation protection measures and equipment. Since intraoperative use of any kind of ionized radiation device is unavoidable in MIS TLIF, the accumulation of radiation exposure to the surgeon, surgical staff, and patient is therefore concerning [10,11,12,13,14,15,16,17]. CAN provides the benefit of allowing the medical team to leave the operating room during image acquisition. Several published studies have reported lower radiation doses to the surgeon and staff when using CAN compared to 2D-fluoroscopy, although at the cost of massively increased radiation exposure to the patient [16,20,31,32,33]. Additionally, CAN still necessitates further 2D-fluoroscopic imaging for the 3D-navigation set up and validation of implants. However, in free-hand MIS TLIF, with the use of correct protective measures, the absorbed radiation can be reduced by up to 98%, which can put the overall radiation exposure to the surgical staff well below the defined levels regardless of the applied imaging technique [10,20,23,34].

In a randomized control trial, we have previously compared MIS TLIF with or without CAN, and we could not demonstrate a statistically significant different level of radiation exposure between the two groups on unprotected areas of the surgeon (effective radiation dose: 41.4 µSv vs. 42.6 µSv). On the other hand, the exposure to radiation of the patient was significantly lower with conventional 2D-fluoroscopy (fluoroscopy time of 64 s vs. 109 s; dose–area product of 526 cGycm^2^ vs. 829 cGycm^2^; total radiation dose of 65 µSv vs. 108 µSv at the neck, 123 µSv vs. 305 µSv at the chest, and 823 µSv vs. 2728 µSv at the umbilical area) [18]. Furthermore, when we assessed the exposure on the dosemeter under the protective lead, all readings were below the technical detection limit for both groups. The effective average radiation dose for the surgeon was 41.4 µSv per segment. This is well below the accepted margin of safety, but also much lower than previous studies performed to assess radiation exposure and MIS TLIF with 2D-fluoroscopy [5]. However, the results of our study should not be easily generalized, since all procedures were performed by experienced surgeons with an essentially completed learning curve and our patient selection excluded lumbar scoliotic deformities and recurrent surgery at the index levels. This implies that our surgical approach may be more appropriately applied by expert spine surgeons who are proficient in performing mono- or bilevel free-hand MIS TLIF procedures. Furthermore, it is important to acknowledge the constraints inherent to our study’s primary focus on the radiation exposure faced by both the surgeon and the patient. As a consequence of this narrowly defined scope, this report does not consider long-term clinical or surgical outcomes.

There are a number of studies that report a higher radiation exposure to the surgeon when using 2D-fluoroscopy versus navigated procedures for spinal instrumentation. In 2019, Pennington and colleagues performed a solid systematic review and meta-analysis of the literature in patients undergoing any image-guided thoracolumbar fusion performed via a posterior approach with different imaging modalities (2D-fluoroscopy, 2D-fluoroscopy with preoperative CT-guided navigation, iCT-guided navigation, and robot-assisted instrumentation). They identified a significantly higher level of radiation exposure to the surgeon with 2D-fluoroscopy (6.0 µSv per screw) compared to 2D-fluoroscopy with preoperative CT-guided navigation (0 µSv per screw) and iCT-guided navigation (3.0 µSv per screw), as well as a relatively high radiation exposure to the patient (260 µSv per screw), although this was lower than 2D-fluoroscopy with preoperative CT-guided navigation (270 µSv per screw) and iCT-guided navigation (1200 µSv per screw) [20]. However, these results fail to address the specific MIS TLIF technique, since their review was based on diverse posterior surgical approaches. For instance, no information about cage implants was provided. Some studies performed cortical bone trajectory screw placement, some screw placements were thoracic, no restrictions of diagnosis were made, and no standardized acquisition method for the radiation exposure was considered. Several studies were also performed on cadavers or anatomic models. Moreover, the reported radiation exposure among the results in the literature was inconsistent, and simple reporting of fluoroscopy times and dose–area products is, in our opinion, insufficient for evaluating the real radiation exposure.

Conversely, our study was very specific in terms of inclusion criteria and surgical technique, and was strengthened by the benefits of a prospective trial. Therefore, we argue that our radiation-sparing 2D-fluoroscopy-guided monosegmental or bisegmental MIS TLIF technique (with or without bilateral decompression and/or cement) is comparable to CAN with regard to radiation exposure to the surgeon, while simultaneously massively decreasing the radiation exposure to the patient.

Another benefit of CAN is its improved pedicle screw placement accuracy [35]. However, these findings, measured mostly in 2 mm pedicle breach increments, is often poorly translated to clinical relevance [25,36,37]. In one systematic review comparing CAN with the free-hand technique, the pedicle screw accuracy for the lumbar region showed no significant difference between the two methods [25]. In our study, we found 97.1% of our pedicle screws to be correctly positioned, with only three screws breaching the pedicle by more than 2 mm, which is in line with previously published results. More importantly, there were no clinical consequences of the pedicle breach. This highlights the safety and accuracy of our radiation-sparing technique.

Another drawback when using CAN is the prolongation of the surgical time during setup of the tracking device and during the scan. In our patient series, an average of approximately 20 additional minutes was required to prepare and perform the intraoperative 3D scan and to set up the navigation (patient tracker placement, 3D scan, referencing of instruments to be navigated, additional wound closure).

Since high acquisition and maintenance costs can be a burden for some hospitals, and only high-volume spine centers benefit from the cost-effectiveness of CAN, its adoption is still limited [38]. In an international survey of spine surgeons conducted in 2013, 66% never used CAN for spinal fusions, and in a recent national survey of spine surgeons in the United States, still only 40% routinely used CAN [28,30]. In a recent systematic review on MIS TLIF, 79% reported the use of 2D-fluoroscopy as the routine imaging modality [30]. To achieve a better outcome, even low-cost modifications to the surgical technique can lead to huge differences. For example, Fan and colleagues [39] showed that through the application of a radio-opaque locator device and a screw assistant device for the application of K-wires and Jamshidi needles, a significant reduction in fluoroscopy time and effective dose exposed to the surgeon could be achieved, as well as improved pedicle screw accuracy. Our radiation-sparing free-hand 2D-fluoroscopy MIS TLIF technique can be realized by instruments and low-cost measures which are mostly already available.

## 5. Conclusions

With the presented advanced radiation protection principles and the radiation-sparing surgical protocol, free-hand MIS TLIF using 2D-fluoroscopy is possible with low radiation exposure to both the surgeon and the patient while simultaneously maintaining a high accuracy.

## Figures and Tables

**Table 1:** Clinical characteristics of free-hand 2D-fluoroscopy-guided MIS TLIF.

**Patient characteristics**			
	Patients		24
	Age (years)		62.4 ± 14.0
	Female/male ratio		12:12
	Body mass index (kg/m^2^)		27.6 ± 5.3
**Characteristics of surgical segments (*n* = 27)**			
	Spondylolisthesis, Meyerding grade I		9
	Spondylolisthesis, Meyerding grade II		16
	Spondylolysis		15
	Segment operated		
		L3–L4	2
		L4–L5	12
		L5–S1	13
**Operative characteristics**			
	Bilateral decompression		11
	Monosegmental stabilization		21
	Bisegmental stabilization		3
	Total pedicle screws		102
	No pedicle breach or pedicle breach ≤2 mm		99 (97.1%)
	Pedicle breach >2 mm		3 (2.9%)
	Estimated blood loss (mL)		256 ± 328
	Operation time (min)		184 ± 52

Values are presented as numbers (%) or mean ± standard deviation.

**Table 2:** Average radiation exposure calculated per segment.

		All per Segment
Number of fluoroscopic images		75 ± 43
Fluoroscopy time (s)		64 ± 34
Dose–area product (cGycm^2^)		526 ± 388
Effective radiation dose (µSv)		
	Surgeon	41.4 ± 11.7
Total radiation dose (µSv)		
	Patient neck	65 ± 40
	Patient chest	123 ± 116
	Patient umbilical	823 ± 862

Average radiation exposure is presented as mean ± standard deviation for 21 monosegmental and 3 bisegmental MIS TLIF calculated per segment. Data on the monosegmental MIS TLIF procedures have already been published previously [18].

## Data Availability

The data presented in this study are available upon request from the corresponding author. The data are not publicly available due to privacy and ethical concerns.
